# Pulmonary arterial hypertension–associated cerebellar impairment extends evidence linking cardiometabolic and neurodegenerative diseases

**DOI:** 10.1172/JCI212006

**Published:** 2026-11-02

**Authors:** Michael W. Schwartz, Karin E. Bornfeldt

**Affiliations:** 1 Division of Metabolism, Endocrinology and Nutrition, Department of Medicine, UW Medicine Diabetes Institute, and; 2 Department of Laboratory Medicine and Pathology, University of Washington, Seattle, Washington, USA.

## Abstract

Growing evidence shows that the risk of cognitive impairment is increased across a range of metabolic and cardiovascular diseases, but underlying mechanisms remain poorly understood. One possibility involves activation of microglia, a glial cell type of the central nervous system with morphological and transcriptional features comparable to resident macrophages in other tissues that plays specialized roles in supporting neuronal and brain function. Combining clinical investigation in pulmonary arterial hypertension (PAH) cohorts with mouse model and microglial cell culture studies, a study by Okawa, Shi, and colleagues in this issue of the *JCI* adds compelling, albeit indirect, evidence linking impaired lysosomal lipid handling in both endothelial cells and microglia to a specific neurological disorder associated with PAH. The findings open opportunities for understanding how cardiopulmonary disease predisposes to neurodegeneration.

## Microglial activation associates with neurological deficits

Cardiovascular diseases and their comorbid disorders, ranging from obesity and diabetes to autoimmune diseases such as rheumatoid arthritis, are associated with both systemic inflammation and an increased risk of cognitive impairment (1–3). Insight into underlying mechanisms has proven elusive, but growing evidence points to a role played by microglia, the macrophage-like glial cell type residing in the brain, in neuroimmune communication contributing to both health and disease (4, 5).

Like macrophages, microglia are highly plastic cells that adopt different phenotypes and functions in a context-dependent manner. In addition to synaptic pruning, maintenance of myelin integrity, and other physiological functions (6), microglia continuously survey the local brain microenvironment for signals arising from tissue damage or invading pathogens. Such stimuli rapidly induce a phenotype switch that enables these cells to digest and phagocytose damaged tissue and invading pathogens. Although often referred to as microglial activation, this term fails to capture recent evidence of a continuum of distinct microglial states across the life span, both in health and in disease (7). Nevertheless, this pro-inflammatory, phagocytic response is eventually supplanted by a shift toward microglial processes that promote tissue repair in cooperation with astrocytes and other glial cell types.

In addition to cues in the local microenvironment, microglial activation can be triggered by systemic inflammation (8, 9), but links between this response and neurodegeneration remain largely speculative. In this issue of the *JCI*, Okawa, Shi, and colleagues add compelling, albeit indirect, evidence of a role for microglial activation in a specific neurological deficit associated with pulmonary arterial hypertension (PAH) (10). Their study combining clinical investigation with mutant mouse models and microglial cell culture studies is a true tour de force. But as is often the case in cutting-edge, innovative research, the reported findings raise as many questions as they answer.

## Cerebellar dysfunction in individuals with PAH

PAH is a serious systemic inflammatory illness characterized by narrowing and stiffening of the pulmonary arteries. The disease appears to result in part from inflammation and impaired lysosomal lipid metabolism by pulmonary artery endothelial cells (11, 12) (Figure 1). In addition, Okawa, Shi, et al. now offer clear evidence linking PAH to degeneration of a neurocircuit that projects from the left cerebellum to the left superior frontal gyrus (the L-CRBL–L-SFG axis) (10). Structural and functional MRI data from a large repository maintained by the UK Biobank show that compared with controls, patients with PAH exhibited reduced cerebellar white matter volume, thinner frontal gyri, and weakened cerebellar-frontal connectivity. These findings were subsequently confirmed in a small cohort of rigorously diagnosed individuals with PAH, and they align with neuropsychiatric features associated with impairments in cerebellar-frontal cortex connectivity: reduced processing speed and working memory, worsened executive function, impaired abstract reasoning, and reduced problem-solving ability.

The simplest explanation for how a disease affecting pulmonary vasculature might cause neurodegeneration is via reduced brain perfusion or oxygenation. Here, however, this possibility seems unlikely, given that this very specific set of neurological deficits is not seen in patients with related cardiopulmonary conditions (10). Thus, other, less straightforward explanations must be considered.

Among these explanations is that PAH and L-CRBL–L-SFG abnormalities arise from a shared underlying disease mechanism, and much of the data that Okawa, Shi, et al. present address this possibility. Central to this hypothesis is that in PAH, oxysterols and related lipid metabolites accumulate in pulmonary artery endothelial cells because of a lysosomal defect. The resulting worsened inflammation and endothelial dysfunction increase pulmonary vascular resistance, leading to pulmonary hypertension (12). Could the neurological manifestations of PAH involve a similar pathogenesis?

## Can lysosomal dysfunction link cardiometabolic disease to neurodegeneration?

This premise of similar pathobiology is supported by evidence linking lysosomal dysfunction to neurodegenerative disorders, including Alzheimer’s disease and Parkinson’s disease (13, 14), as well as to atherosclerotic cardiovascular disease (15, 16). To further investigate this possibility, the authors take an innovative approach: they leverage published evidence linking PAH severity to a single nucleotide polymorphism that reduces expression of NCOA7 (12), a protein that facilitates lysosomal lipid handling. Several prior observations support the hypothesis that upregulation of NCOA7 plays a protective role in patients with PAH. For one, the authors previously reported that in a mouse model of PAH, created by overexpression of IL-6 in airway epithelial cells (17), NCOA7 deficiency exacerbated lysosomal dysregulation, elevating generation of oxysterols and inflammatory molecules in pulmonary arterioles and endothelial cells (12). They next considered whether NCOA7 deficiency might also exacerbate the neurological deficits associated with PAH.

They report that in patients with PAH, circulating inflammatory mediators (identified using Olink plasma proteomics) associate negatively with a single nucleotide polymorphism in the *NCOA7* gene that increases its expression. This same polymorphism associates with improved L-CRBL–L-SFG connectivity in individuals with PAH, again consistent with a protective role played by NCOA7.

To further interrogate this link, Okawa, Shi, et al. returned to the mouse model of PAH (originally described in ref. 17). They report that as they observed in humans, cerebellar degeneration occurred in these mice and that its severity was also increased by NCOA7 deficiency. Combined with evidence that cerebellar NCOA7 expression is increased in both humans and mice with PAH, these findings suggest that (a) NCOA7 upregulation helps limit oxysterol-induced damage and (b) reduced NCOA7 activity is a “second hit” that exacerbates disease severity.

What is the nature of this second hit? NCOA7 binds to the cytoplasmic domain of the vacuolar ATPase proton pump (18), helping maintain the low lysosomal pH needed for proteases to digest proteins and for lysosomal acid lipase to digest lipids (Figure 1). Loss of NCOA7 therefore leads to lysosomal alkalinization and trapping and oxidation of lipids, with subsequent release of oxysterols into the local microenvironment and, ultimately, into the circulation. Loss of NCOA7 has also been shown to reduce expression of lysosomal markers in brain and to cause neuronal patterning defects in animal models (18). The second hit induced by NCOA7 deficiency, therefore, could affect the brain as well as vascular endothelium (19).

To determine if NCOA7 deficiency predisposes to microglial activation, the authors employed a cultured human microglial cell line. They report that in these cells, NCOA7 silencing induced lysosomal dysfunction, increased intracellular oxysterol accumulation, impaired phagocytosis, and increased expression of inflammatory cytokines (10), responses that overlap with those associated with NCOA7 deficiency in pulmonary arteriolar endothelium (12) and are consistent with evidence identifying pulmonary oxysterol and bile acid metabolites as predictors of mortality in humans with PAH (20).

If the neurodegenerative consequences of PAH involve impaired lysosomal oxysterol handling by cerebellar microglia, a key question remains: why should this microglial activation response be specific to the cerebellum? One possibility is based on evidence that compared with microglia in other brain areas, cerebellar microglia are endowed with several distinctive features. Among those is heightened responsivity to immunogenic stimuli (21), raising the possibility that cerebellar microglia are uniquely sensitive to mishandling of lysosomal oxysterol metabolism. If true, this attribute would provide crucial support to the hypothesis that PAH and L-CRBL–L-SFG connectivity deficits share underlying disease mechanisms.

## Concluding remarks, key questions, and future directions

Oxysterols have long been implicated in cardiovascular disease, but the extent to which they play a causal role is unresolved. Although the data presented support a model in which PAH-associated neurodegeneration is driven by lysosomal dysfunction in cerebellar microglia, many questions remain unanswered. Are these microglia selectively activated by oxysterols and/or bile acid metabolites? If so, does this activation involve a cell-autonomous mechanism, or do circulating oxysterols play a role? Does oxysterol-induced blood-brain barrier damage contribute to neurodegeneration by increasing microglial exposure to circulating inflammatory mediators? Mouse models in which NCOA7 is deleted selectively from either microglia or endothelial cells will help address these questions.

Finally, can lysosomal protection and reduced intracellular oxysterol accumulation be targeted as a therapeutic strategy in PAH and associated neurodegeneration? That we are now able to ask this question highlights the translational significance of work that has fundamentally advanced our understanding of this devastating illness (10). Insight into underlying mechanisms may ultimately lead to novel approaches to the treatment of PAH and may apply more broadly to understanding the connection between vascular disease and dementia.

## Manuscript information

### AI disclosure.

The authors declare that no AI tools were used in the original research for or writing of this article.

### Conflict of interest.

MWS is a consultant for Olio Labs, Lumen Biosciences, and Gallant Bio. KEB serves on the scientific advisory board of Esperion Therapeutics.

### Funding.

This work is the result of NIH funding, in whole or in part, and is subject to the NIH Public Access Policy. Through acceptance of this federal funding, the NIH has been given a right to make the work publicly available in PubMed Central.

- MWS’s laboratory by NIH National Institute of Diabetes and Digestive and Kidney Diseases grants R01DK083042 and R01DK101997.
- KEB’s laboratory by R35HL184266, P01HL151328, and U01DK142249 from the NIH.

## Figures and Tables

**Figure 1:**
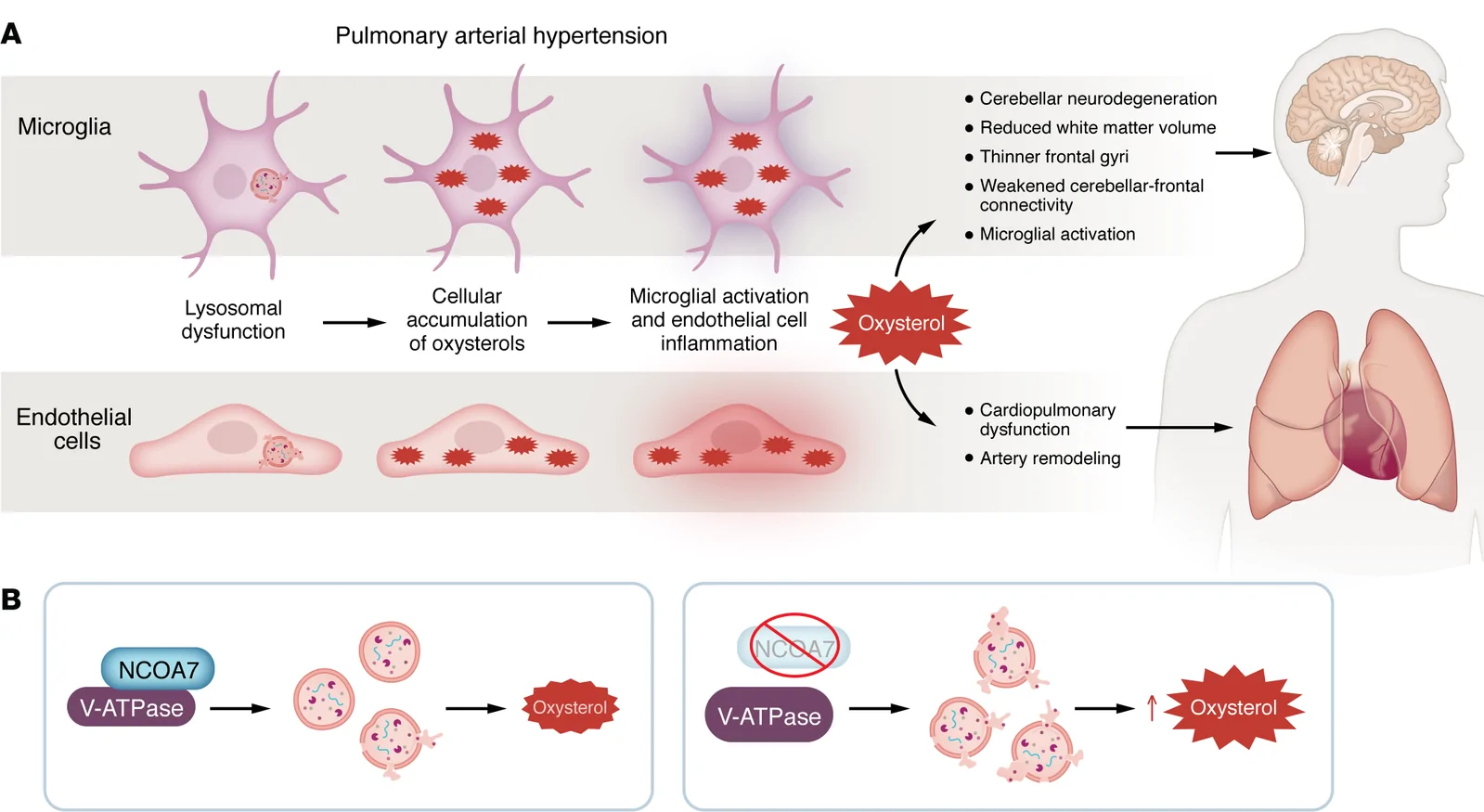
Is lysosomal dysfunction–induced oxysterol generation a shared causal link between pulmonary artery hypertension and neurodegeneration? Pulmonary arterial hypertension (PAH) is caused by pulmonary artery narrowing and stiffening. Research by Okawa, Shi, and colleagues (10) showed that in addition to cardiopulmonary dysfunction, PAH is associated with cerebellar neurodegeneration. (**A**) Mechanistically, their work suggests a common pathogenesis involving lysosomal dysfunction in both endothelial cells and microglia. (**B**) In PAH, impaired lysosomal acidification by vacuolar ATPase (V-ATPase) increases oxysterol generation, exacerbating local and systemic inflammation. This effect is limited by upregulation of nuclear receptor coactivator 7 (NCOA7), which binds one of the V-ATPase subunits. Consequently, NCOA7 deficiency is associated with more severe PAH.

## Version 1 11/02/2026

Electronic publication
